# An Effective Sol-Gel-Functionalized Polyurethane Foams Solid Platform Packed Minicolumns for Complete Extraction of Chromium (VI) from Water: Kinetic, Sorption Isotherms, Thermodynamic Study, and Analytical Utility

**DOI:** 10.1155/2024/3152894

**Published:** 2024-08-06

**Authors:** Effat A. Bahaidarah

**Affiliations:** Department of Chemistry, Faculty of Science, King Abdulaziz University, P.O. Box 80203, Jeddah 21589, Saudi Arabia

## Abstract

In the modern era, sol-gel plays a key role in the progress of a new generation of dispersive solid-phase microextractors (d–*µ* SPMEs) for the removal of organic and inorganic pollutants in complex matrices. Thus, the current study reports the use of sol-gel-functionalized polyurethane foams (PUFs) as a novel solid platform for complete extraction of chromium (VI) species from aqueous media. The planned protocol was based upon the complete extraction of the formed binary complex ion associates between the protonated ether and/or urethane groups of PUFs and chlorochromate anion [CrO_3_Cl]^−^_aq_ in aqueous HCl (≥1M) medium in addition to H-bonding and the electrostatic *π*–*π* interaction that resulted between the CrO_3_Cl^−^ and the silanol group (Si/ZrO_2_, Si–O–Zr) and siloxane (Si–O–Si) groups of the sol-gel. The impact of the analytical parameters (solution pH, natural mineral acids, shaking time, temperature, and chromium (VI) concentrations) was critically studied. At the optimal conditions, the uptake capacity of the established extractor (9.9 mg·g^−1^) was in agreement with the Langmuir adsorption capacity (12.08 mg·g^−1^) of the monolayer. The sorption data fitted well with the pseudo first-order kinetic model (*R*^2^ = 0.9961) with an overall rate constant (*k*_1_) of 0.081 min^−1^ and an equilibrium capacity (*q*_*e*_) of 8.6 mg·g^−1^, which is in a good agreement with the experimental value (9.9 mg·g^−1^). The sorption of the oxyion [CrO_3_Cl]^−^_aq_ onto the solid sorbent is an endothermic and spontaneous process as reflected from the values of ΔH (6.99 kJ·mol^−1^) and Δ*G* (−8.14 kJ·mol^−1^ at 293 K), respectively. The Δ*S* value (15.13 kJ·mol^−1^·K^−1^) reflects that the [CrO_3_Cl]^−^_aq_ retention onto the sol-gel-treated PUFs sorbent proceeded in a more unplanned fashion. Sol-gel-treated PUFs sorbent-packed minicolumns were successfully used for the complete removal of trace levels of chromium (VI) species from water samples. Sorbed chromium (VI) species were recovered with NaOH (0.5 M) and analysed by spectrophotometry, which supports the utility of the sol-gel-treated PUFs as a low-cost solid extractor for water treatment.

## 1. Introduction

Chromium exists in nature in two oxidation forms mainly chromium (III) and chromium (VI) as a liquid, solid, or gas [1] and could reach the aquatic environment from different natural sources and industrial activities [2–4]. Chromium (VI) species such as chromate and/or dichromate ions in water have genotoxic and mutagenic effects on biological systems, while trivalent chromium plays a significant role in the metabolism of humans and retains much lower toxicity [5, 6]. Thus, chromium (VI) in the aquatic environment represents one of the key contaminants; the US-EPA has classified chromium as a group “A” carcinogenic agent of anthropological toxins [4, 7]. Exposure to high levels of chromium (VI) causes breathing problems [7, 8], allergic reactions, ulceration of the skin, and damage to the eyes, liver, kidneys, circulatory and nerve tissues, as well as immune systems [9–11]. On the other hand, chromium (VI) toxicity from food remains a great concern [12]. According to US-EPA and WHO, the maximum allowable edges of chromium (VI) in drinking water are 0.1 *µ*g/mL and 0.05 *µ*g/mL, respectively [13].

A series of analytical techniques for trace determination and preconcentration of chromium species in particular chromate and/or dichromate has been reported [14–22]. The most common spectroscopic techniques are spectrofluorimetry, spectrophotometry [18–20, 22] atomic absorption spectrometry (AAS) [23–26], inductive coupled plasma-mass spectrometry (ICP-MS) [27, 28], atomic emission spectrometry (AES) [29, 30], and total reflection X-ray fluorescence spectrometry (TRXRFS) [31]. Moreover, chromatographic separation and determination involving high-performance liquid chromatography (HPLC) [32–37] and gas chromatography (GC) [8] have also been applied for the trace determination of chromium species in complex matrices. However, complications exist at trace and ultratrace levels with direct quantification of chromium; thus, the enrichment step is compulsory compared to traditional tools with time-consuming approaches, use of large volumes of solvents, and unsatisfactory recovery percentage [33, 34].

Nowadays, the extraordinary development in the use of dispersive solid-phase microextractors (d–*µ* SPMEs) has attracted many researchers [38, 39]. This tool offers a widespread range of applications besides its cost effectiveness, ease of the process, low solvent consumption, high recovery, and rapidity [38]. Solid phase extraction (SPE) and/or solid phase microextraction (SPME) represent one of the most effective techniques that are frequently applied for trace and ultratrace determination of dyes and metal ions in environmental water samples [40–43] and removing pollution from water [44], whether in batch or column (flow) mode. Sponge solid-phase extraction involving the use of polyurethane foams (PUFs) of polyether and/or polyester types as a flexible membrane-like structure has received considerable attention in the last few decades [45, 46]. PUFs have been used as an outstanding substrate for solid-phase extraction for separation and/or preconcentration procedures for trace quantification of various species such as organic and inorganic oxyanions and metal ions [45, 46]. In PUFs, the occurrence of polar and nonpolar groups enhances the analyte sorption [47, 48]. PUFs have several advantages such as chemical stability, availability, and affordability; moreover, they can easily be reused for dyes and other organic pollutants removal desorption [49, 50]. PUFs are also beneficial in column procedures in off-line or flow injection preconcentration systems, since this class of solid-phase extractors has minimum resistance for fluid passage and does not cause over pressure or swelling like other sorbents [46]. The large surface area of nanoparticles could enhance the sensitivity of the solid sorbents and modify their properties [51, 52] by coupling them together in different forms [53–57]. The sol-gel technology with the advantages of optical transparency and chemical robustness has received considerable interest in various applications in the last few years [58–60]. Hence, the current study aimed to (i) synthesize and characterize the sol-gel physically impregnated PUFs as a solid microextractor; (ii) study the retention profile of chromium (VI) from the aqueous by the established sol-gel-treated PUFs solid platform; (iii) properly assign the kinetics, thermodynamics, sorption models, and multimode sorption mechanisms of chromium (VI) by the developed sol-gel-treated PUFs; and (iv) finally, test the analytical utility of the developed microextractor in flow (packed column) and pulse modes for complete extraction and recovery of trace levels of chromium (VI) in environmental water samples.

## 2. Experimental Setup

### 2.1. Reagents, Chemicals, and Materials

All the chemicals and solvents were analytical-reagent grade (AR) and used as received. The chemicals, NaOH, HCl, H_2_SO_4_, HNO_3_, K_2_Cr_2_O_7_, K_2_CrO_4_, and organic solvents such as acetone and isopropanol were purchased from Merck company and were used as received. The precursors methyltrimethoxysilane (C_4_H_12_O_3_Si), methyltriethoxysilane (C_7_H_18_O_3_Si), tetramethyl orthosilicate (C_4_H_12_O_4_Si), tetraethyl orthosilicate (C_8_H_20_O_4_Si), and 3-(trimethoxysilyl)propyl methacrylate (C_10_H_20_O_5_Si) were purchased from Aldrich (Assay > 98). 3-methacryloxypropyltrimethoxysilane (MAPTMS, assay 99% in methanol, Sigma-Aldrich), zirconium (IV) n-propoxide (ZPO, assay 70% in propanol, Sigma-Aldrich, Ireland), and methacrylic acid (MAAH, C_4_H_6_O_4_, assay > 98%, Sigma-Aldrich, Ireland) have been used in sol-gel preparation. Low density polyethylene (LDPE) bottles, Nalgene, were used for storage of water samples. Commercial open-cell polyether-type-based PUFs, which were supplied locally in Saudi Arabia, were cut into cubes of approximately one cm^3^, washed with 8% HCl and deionized water until the washing solutions were free from chloride ions [61–63], and then washed with acetone to remove organic contaminates and finally dried in an oven at 80°C for 2 h. A series of standard solutions (0.005–0.01% m/v) of the ion-pairing reagent procaine hydrochloride (PQ^+^.Cl^−^) was prepared individually in deionized water. Britton–Robinson (B–R) buffer solutions of pH 2–11.7 were prepared as reported earlier [64].

### 2.2. Instrumentation

A scanning electron microscope (SEM; JSM-5910, JEOL) was used for recording the surface morphology of the synthesized sol-gel and sol-gel-treated polyurethane foams (PUFs). An energy-dispersive X-ray spectroscopy (EDX) was used to confirm the elemental composition. Scanning atomic force microscopy (AFM; Pacific Nanotechnology: Nano-RTM) was also used to fully characterize the surface topography and roughness of the prepared sol-gel samples. FTIR spectra of the sol-gel and sol-gel-treated PUFs were recorded using a JASCO-430 model spectrometer. PerkinElmer's inductively coupled plasma-optical emission spectroscopy (ICP-OES), Optima (California, CT, USA), was used for chromium determination at the optimum operational parameters (Electronic Supplementary Information (ESI.1, Table 1)) as a method validation. A UV-Vis spectrometer (Shimadzu UV-Vis 1800, Japan) was used for recording the electronic spectra and the absorbance (at 355 nm) of chromium (VI) solutions before and after extractions in 10.0 mm quartz cells. A corporation precision scientific mechanical shaker (Chicago, CH, USA) and a thermostatic controlled shaker (GFL-1083 model, Germany) were used in batch experiments. Chromatographic minicolumn packed extraction was performed on the solid-phase extraction manifold system of Agilent Technologies, 1200 USA. A pH meter (inoLab pH/ion level 2) and a Milli-Q Plus system (Millipore, Bedford, MA, USA) were used for pH measurements and for providing ultrapure water, respectively.

### 2.3. Preparation of Sol-gel

**Scheme 1:**
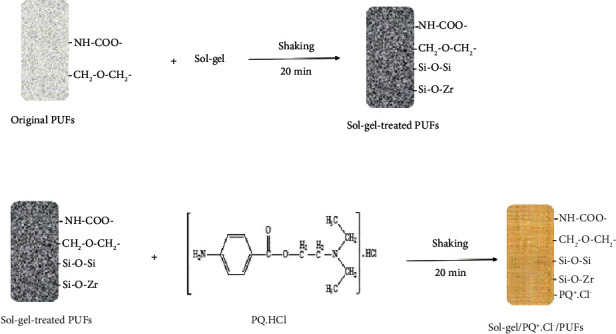
A scheme describing the preparation of the proposed sol-gel/PQ^+^.Cl^−^/PUFs solid extractor.

In this preparation, the mixture of the two hybrid precursors was used to form stable and homogenous sol-gel. The first precursor is an organically modified silicon precursor (MAPTMS), and the other one is an organically modified zirconium complex which was prepared from the complex formation of ZPO by MAAH, as reported earlier [53]. During the initial synthesis, the relative proportions of the MAPTMS : ZPO were 80 : 20 and the theoretical hydrolysis degree was 50% against the total content of reactive alkoxide groups. The synthesis process was carried out in three sequential steps. First, two simultaneous reactions were performed for 45 min. The first reaction was the prehydrolysis of MAPTMS by using an aqueous HNO_3_ solution (0.1 M) with a 4 : 1 ratio. MAPTMS was immiscible with water for 5 min, and then all species became miscible due to methanol production. The parallel reaction involved chelating ZPO using MAAH to block alkoxide groups and to minimize the precipitation. The second main step was slowly adding the partially hydrolysed MAPTMS to the zirconate complex. Finally, after 5 min, neutral hydrolysis of the mixture was completed by adding deionized water gently [53]. The overall preparation of the sol-gel material has been reported by MacHugh [73], and the experimental steps are depicted in ESI.2, scheme 1.

### 2.4. Preparation of the Sol-gel/PQ^+^.Cl^−^ Functionalized PUFs Sorbent

The sol-gel-functionalized polyurethane foam (PUFs) sorbent was fabricated as follows:

1. The dried PUFs cubes (about 2.0 g) were shaken with the sol-gel dissolved in isopropanol (IPA, 25 mL, 5% v/v) with efficient stirring for 20 min to physically impregnate sol-gel onto the PUFs cubes as reported [62].
2. The sol-gel-treated PUFs (sol-gel/PUFs) cubes were separated out by decantation and pressed between two sheets of filter paper to remove any unbound sol-gel from the PUFs.
3. The dried sol-gel-treated PUFs (about 2.0 g) were then shaken with procaine hydrochloride (0.005–0.01% m/v) with efficient stirring for 20 min. The sol-gel-/PQ^+^.Cl^−^ -treated PUFs cubes (sol-gel/PQ^+^.Cl^−^/PUFs) were separated out by decantation. Finally, the sol-gel/PQ^+^.Cl^−^/PUFs cubes were pressed between two sheets of filter paper to remove any traces of excess PQ^+^.Cl^−^, as reported earlier [62].

A schematic diagram describing the preparation of the proposed sol-gel/PQ^+^.Cl^−^/PUFs solid extractor is illustrated in Scheme 1.

### 2.5. Static Experiments

A precise mass of sol-gel/PQ^+^.Cl^−^/PUFs cubes (0.05–0.1 g) was shaken for 50 min with an aqueous solution (100 mL) of chromium (VI) at various known concentrations (0.05–50.0 *µ*g·mL^−1^) in HCl (1.0 M) in a conical flask (200 mL) at 25 ± 1°C on a mechanical shaker. After equilibration, the aqueous phase was separated out by decantation, and the amount of chromium (VI) remaining in the aqueous phase was determined spectrophotometrically from its absorbance at 355 nm (*λ*_max_) *versus* reagent blank. The retained chromium (VI) species on the established extractor were then calculated from the difference (*A*_*b*_ − *A*_*f*_) of the absorbance before (*A*_*b*_) and after (*A*_*f*_) extraction.

Similarly, the impact of various parameters (pH solution, shaking time, and temperature) was critically studied, and the distribution ratio (*D*) and extraction percentage (%*E*) of chromium (VI) uptake by the established extractor were determined as reported [62].

### 2.6. Analytical Applications

#### 2.6.1. Extraction and Recovery of Chromium (VI) from Deionized and Environmental Tap Water were Performed by Minicolumn (Flow) Mode of Separation

A series of deionized water and prefiltered tap water samples (100 mL) adjusted to pH ≤ 1 using HCl (1.0 M, 10 mL) and spiked with known concentrations of chromium (VI) (0.01–1.0 *µ*g/mL) were passed individually through sol-gel/PQ^+^.Cl^−^/PUFs (0.4 ± 0.002 g) packed minicolumn at 10 mL·min^−1^ flow rate. Complete retention of chromium (VI) took place as indicated from the absorbance at *λ*_max_ 355 nm and/or ICP-OES measurements of chromium (VI) in the effluent against the reagent blank. A 2.0 mL solution of NaOH (0.5 M) was used as an eluting agent for the complete recovery of chromium (VI) from the sol-gel/PQ^+^.Cl^−^/PUFs packed column at 0.5 mL·min^−1^ flow rate. The absorbance at *λ*_max_ 355 nm and/or ICP-OES signal intensity of the fractions of the eluate were measured against the reagent blank. The %*E* and *D* values were computed, whereas the recovery percentage (%*R*) was calculated by using the following equation:

(1) $$\begin{array}{c} \%R=\frac{\text{eluted}}{\text{added}}\times 100. \end{array}$$

#### 2.6.2. Extraction and Recovery of Chromium (VI) from Environmental Water by Pulse Mode of Separation

A series of 100 mL of prefiltered tap water samples spiked with known concentrations of chromium (VI) (0.01–1.0 *µ*g/mL) adjusted to pH ≤ 1.0 with HCl (1.0 M, 10 mL) was transferred into a conical flask (200 mL). Equal weights (0.2 ± 0.001 g) of the established sorbent sol-gel/PQ^+^.Cl^−^/PUFs were placed in the medical syringes (20 mL, capacity) as pulse columns individually. The columns were pulsated 25–30 times through chromium (VI) solutions of the tap water. The sorbed chromium (VI) was finally recovered with NaOH (20 mL, 0.5 M) after 25–30 pulses as indicated from the absorbance at 355 nm (or ICP-OES) measurements of the recovered chromium (VI). The %*R* of the chromium (VI) species was finally computed using equation (1).

## 3. Results and Discussion

The development of a dispersive solid-phase extractor involving the use of sol-gel/PQ^+^.Cl^−^/PUFs as an ideal dispersive solid-phase microextractor for chromium (VI) removal from aqueous is a great concern. Thus, chromium (VI) sorption from aqueous media of pH < 1 by sol-gel/PQ^+^.Cl^−^/PUFs as a dispersive solid-phase extractor (d–*µ* SPME) was the primary study. Significant chromium (VI) uptake (>75%) onto the modified PUFs sorbent was easily achieved compared to untreated PUFs (20–25%). Thus, in the next study, sol-gel-treated PUFs were successfully used as a d–*µ* SPME for chromium (VI) removal and subsequent recovery from the test aqueous media.

### 3.1. Characterization of Sol-gel

#### 3.1.1. Surface Morphology Characterization

Scanning electron microscope (SEM) image of the prepared hybrid sol-gel (ESI.3) displayed uniform shapes of the surface morphology of the nanohybrid sol-gel as reported earlier [55, 74]. Atomic force microscopy (AFM) of the hybrid sol-gel was recorded further for initial evaluation of the surface roughness. The AFM images (ESI.4) showed surface roughness, the nanostructure of the sol-gel, as well as support the suitability of the sol-gel as a nanoparticle to be chemically impregnated on the solid support-based PUFs surface and membrane substrate [53]. Furthermore, the energy-dispersive X-ray spectroscopy (EDX) analysis of the hybrid sol-gel was performed to identify the presence of the main elements (ESI.5). The data show the formation of nanosized hybrid sol-gel and the appearance of the main elements Si, Zr, and O in uniform distribution with high homogeneity of the material.

#### 3.1.2. Structural Characterization

The FTIR spectrum for the prepared sol-gel in the range 500–4000 cm^−1^ is shown in ESI.6A. The data revealed that the characteristic vibrations at 1000, 1100, and 1250 cm^−1^ corresponding to Si–O–Zr, Si–O–Si, and –C=O were in good agreement with the data reported earlier [53–55]; whereas the observed vibrations at 2900 and 3450 cm^−1^ were safely assigned to C–H and Si–OH, respectively. In the range 850–1250 cm^−1^ (ESI.6B), the spectrum of the sol-gel also displayed the characteristic vibrations at 900, 950, 1100–1125, and 1200 cm^−1^, which were safely assigned to Si–OH, Si–O–Zr, Si–O–Si, and Si–O–C, respectively [53–55]. The FTIR spectrum of the sol-gel also displayed the presence of the same chemical vibrations of the precursor and the prepared materials [53, 54]. The observed broad stretching vibration in the range of 800–1200 cm^−1^ was safely assigned to the silicate network which is combined of the silanol stretches (Si–OH: 890 cm^−1^) and Si–O–Si and Si–O–Zr vibrations (840 and 1010–1050 cm^−1^) and (940 cm^−1^), respectively [53–55], whereas the Si–O–C in the methoxy-silane group in MAPTMS could be responsible for the stretching vibration at 1170 cm^−1^ [53]. The vibrations at 1635 and 1533 cm^−1^ were assigned to the symmetric (*ν*s) and asymmetric (*ν*as) vibrations of the carboxylic group (COO−), respectively, [55, 56] with Δ*v* (COO−) of 10^2^ cm^−1^ in a bidentate fashion in Zr–MAAH complex.

### 3.2. Optimization of the Analytical Parameters

**Figure 1:**
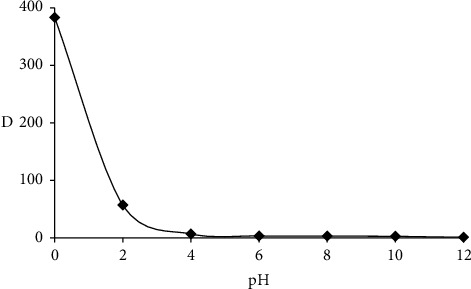
Plot of chromium (VI) retention onto the established sol-gel/PQ^+^.Cl^−^/PUFs sorbent *versus* pH at 25 ± 1°C after 60 min shaking time.

The impact of various parameters that control the analytical utility of the established sol-gel/PQ^+^.Cl^−^/PUFs solid microextractor towards chromium (VI) removal from aqueous media and sequential determination was critically studied in more detail using batch and flow modes of separation. The pH controls the surface charge of the solid-phase extractor and the analyte dissociation and/or ionization on the active sites of the adsorbent [22, 75]. Thus, the influence of pH on chromium (VI) sorption by the sol-gel-modified PUFs was critically studied in different pH solutions (pH ≈ 1.0–12.0). The results are illustrated in Figure 1 and show that the maximum chromium (VI) retention was achieved at a pH < 1.0, where chromium (VI) is highly extracted since the quantity of HCrO_4_^−^ ions is directly proportional to the quantity of the acid and the complex ion associate formed on/in the sol-gel-treated PUFs [22, 75]. When growing the solution in pH > 1, chromium (VI) retention markedly decreased and reached a minimum value since the analyte is present in a highly polar form and the solid-phase is most likely negatively charged [22, 75]. At high pH ≥ 1.5, the formation of nonextractable chromium (VI) species and/or hydrolysis of the complex ion associate are most likely predominant [22, 75].

Chromium (VI) oxyions occur as negatively charged species and are rapidly hydrolysed forming various species (neutral, anionic, or oxyanions) conditional on the pH of the aqueous [76]. At pH < 1.0, the chemical equilibria of oxychromium (VI) species can be stated as follows [75, 76]:

(2) $$\begin{array}{c} 2H_{2}{\text{CrO}}_{4}⇌2{\text{HCrO}}_{4}^{-}+2H^{+}⇌{\text{Cr}}_{2}O_{7}^{2-}+H_{2}O \end{array}$$

The impact of the presence of a series of mineral acids individually such as HCl, H_2_SO_4_, HNO_3,_ and HClO_4_ (1.0 M) was critically studied. The sorption of oxychromium (VI) by the proposed dispersive extractor increased in the following order:

(3) $$\begin{array}{c} \text{HCl }>{\text{HClO}}_{4}>H_{2}{\text{SO}}_{4}>{\text{HNO}}_{3} \end{array}$$

In HCl media, chromium (VI) is present as a chlorochromate (CrO_3_Cl^−^) anion [75, 77], which is directly proportional to the formation and extraction of the complex ion associated in the organic phase as

(4) $$\begin{array}{c} {\text{HCrO}}_{4}^{-}+\text{HCl}⇌{\text{CrO}}_{3}{\text{Cl}}^{-}+H_{2}O \end{array}$$

(5) $$\begin{array}{c} {\text{CrO}}_{3}{\text{Cl}}_{\text{aq}}^{-}+{\text{PQ}}^{+}.{\text{Cl}}_{\text{PUFs}}^{-}⇌{\left[{\text{PQ}}^{+}.{\text{CrO}}_{3}{\text{Cl}}^{-}\right]}_{\text{PUFs}} \end{array}$$

At a low pH and in the presence of HCl (1.0 M), the available chelating sites ether oxygen (–CH_2_–O–CH_2_–) and/or urethane nitrogen (–NH–COO–) linkages in the PUFs sorbents membrane are protonated resulting in high chromium (VI) sorption retention as follows [62]:

With the ether oxygen group of PUFs as

(6) $$\begin{array}{c} {\left(–{\text{CH}}_{2}–O–{\text{CH}}_{2}–\right)}_{\text{PUFs}}+H^{+}⇌{\left(–{\text{CH}}_{2}–{\text{HO}}^{+}–{\text{CH}}_{2}–\right)}_{\text{PUFs}} \end{array}$$

(7) $$\begin{array}{c} {\left(–{\text{CH}}_{2}–{\text{HO}}^{+}–{\text{CH}}_{2}–\right)}_{\text{PUFs}}+{\left({\text{CrO}}_{3}{\text{Cl}}^{-}\right)}_{\text{aq}}{⇌\left[\left(–{\text{CH}}_{2}–{\text{HO}}^{+}–{\text{CH}}_{2}–\right)\left({\text{CrO}}_{3}{\text{Cl}}^{-}\right)\right]}_{\text{PUFs}} \end{array}$$

With the urethane nitrogen group of PUFs as

(8) $$\begin{array}{c} {\left(–\text{NH}–\text{COO}–\right)}_{\text{PUFs}}+H^{+}⇌{\left(–{\text{NH}}_{2}^{+}–\text{COO}–\right)}_{\text{PUFs}} \end{array}$$

(9) $$\begin{array}{c} {\left(–{\text{NH}}_{2}^{+}–\text{COO}–\right)}_{\text{PUFs}}+{\left({\text{CrO}}_{3}{\text{Cl}}^{-}\right)}_{\text{aq}}{⇌\left[\left(–{\text{NH}}_{2}^{+}–\text{COO}–\right).\left({\text{CrO}}_{3}{\text{Cl}}^{-}\right)\right]}_{\text{PUFs}} \end{array}$$

Thus, in the subsequent study, the HCl concentration in the extraction media of chromium (VI) was adopted at 1.0 M.

The impact of shaking time (0.0–90 min) on chromium (VI) uptake from aqueous media at the optimized pH by the established solid sorbent was studied. The results (ESI.7) revealed fast retention of the chromium (VI) by the established sorbent, and the equilibrium was attained within 50 min of shaking. The available active binding sites and the surface area of the microextractor sorbent are high at the early shaking time. Thus, minimizing the equilibrium time of the SPE towards chromium (VI) sorption is expected. Therefore, it can be concluded that the chromium (VI) partitioning ratio between the sol-gel-modified PUFs and the test aqueous solution at pH < 1 is high, and that the sorbent has excellent performance. Thus, a 50 min shaking time was adopted in the subsequent experiments and the results suggested the use of the sol-gel-treated PUFs as the solid-phase extractor in packed columns.

The outcome of temperature (20–50°C) of the test aqueous solution on chromium (VI) sorption by the established sol-gel/PQ^+^.Cl^−^/PUFs microextractor at the optimized pH and shaking time was studied. The distribution factor *D* (ESI.8) of chromium (VI) increased on growing temperature, revealing the endothermic characteristics of analyte uptake by the established dispersive solid-phase microextractor. Moreover, growing temperature may also reduce the number of water molecules available to solvate the chromium (VI) oxyions in the extraction media at the optimized pH, which would therefore be forced out of the solvent phase into the sorbent. Some free water molecules are also favourably unconfined from the hydration sheets around the chromium (VI) species on increasing temperature, resulting in enhanced extraction [22, 78].

The influence of chromium (VI) over a wide range of equilibrium concentrations (0.0–70 *µ*g/mL) onto the established sol-gel/PQ^+^.Cl^−^/PUFs solid sorbent at the optimal parameters of pH < 1 and shaking time (50 min) was studied. The plot of *D* values *versus* their initial concentrations in the bulk aqueous solution (ESI.9) displayed a maximum *D* value of chromium (VI) sorption onto the established solid dispersive extractor from diluted and/or moderate chromium (VI) levels. The *D* value decreased on growing chromium (VI) concentration, where the solid sorbent membranes and surface area became more saturated with the sorbed oxychromium (VI) ions. The quantity of sorbed chromium (VI) on the sol-gel/PQ^+^.Cl^−^/PUFs sorbent varied more/or less linearly at a low or moderate analyte concentration in the aqueous HCl solution.

### 3.3. Sorption Isotherm Study of Chromium (VI)

**Figure 2:**
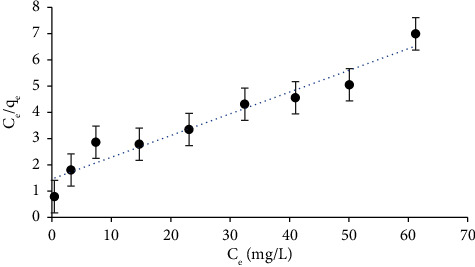
Plot of *C*_*e*_/*q*_*e*_*versusC*_*e*_ (Langmuir isotherm model) at 25 ± 1°C.

The uptake capacity (*q*_*e*_) was calculated by using the following relationships:

(10) $$\begin{array}{c} q_{e}=\frac{\left({\;C}_{o}-C_{e}\right)\;V}{m}, \end{array}$$

where *C*_*ο*_ and *C*_*e*_ are the initial and residual chromium concentrations (mg·L^−1^), *V* is the volume of a chromium (VI) solution (*L*) and *m* is the weight of the adsorbent (*g*). The maximum *q*_*e*_ was found to be 9.9 mg·g^−1^ at 60 *μ*g·mL^−1^ initial concentration. More importantly, Langmuir and Freundlich isotherm models were applied to describe the adsorption process of Cr (VI) onto the treated PUFs. The Langmuir isotherm model indicates that a monolayer of the analyte was formed on the surface of the homogenous adsorbent, which can be demonstrated as follows:

(11) $$\begin{array}{c} \frac{C_{e}}{q_{e}}=\frac{1}{q_{m}K\iota }+\frac{1}{q_{m}}C_{e}, \end{array}$$

where *C*_*e*_ (mg·L^−1^) is the residual concentration at equilibrium and *Kl* (L·mg^−1^) and *q*_*m*_ are the Langmuir constants which represent the adsorption-free energy and the maximum amount of capacity adsorption of the monolayer (mg·g^−1^) of treated PUFs. The plot of *C*_*e*_*/q*_*e*_*versusC*_*e*_ was linear (Figure 2) with a correlation coefficient (*R*^2^) of 0.935. The computed values of *q*_*m*_ and *Kι* were 12.08 mg·g^−1^ and 0.568 L·mg^−1^, respectively. The maximum adsorption capacity (*q*_*m*_) of the established sol-gel/PQ^+^.Cl^−^/PUFs extractor towards chromium (VI) was favourably compared with some of the reported solid adsorbents [79, 80]. The results are summarized in Table 1. The capacity of the developed extractor is somewhat lower compared to other extractors; however, the time consumed to reach equilibrium and the cost of the developed extractor are much lower than other extractors. Thus, in the subsequent study, great attention has been oriented towards the sol-gel-modified solid-phase extractor.

The data were further subjected to the Freundlich isotherm model, which predicts the multilayer adsorption on a heterogeneous system as

(12) $$\begin{array}{c} \text{loq }q_{e}=\log\;K_{F}+\frac{1}{n}\log\;C_{e}, \end{array}$$

where *C*_*e*_ represents the Cr (VI) concentration at equilibrium (mg·L^−1^) and *q*_*e*_ is the amount of Cr (VI) that is adsorbed onto treated PUFs (mg·g^−1^). *K*_*f*_ (mg·g^−1^) and *n* are Freundlich constants that were obtained from the linear plot of log *q*_*e*_ versus log *C*_*e*_ and help in determining the capacity and intensity of the adsorption, respectively. *K*_*f*_, which can be defined as the adsorption or distribution coefficient and exemplifies the quantity of chromium adsorbed onto modified PUFs for a unit equilibrium concentration, was found to be 0.897, while the calculated *n* value (1.65) gives the nonlinearity degree between the solution concentration and the adsorbent. If *n* = 1, the adsorption is linear; if *n* < 1, this suggests that the adsorption process is chemical; if *n* > 1, this means that the physical adsorption is satisfactory [81].

According to the modelling, the static adsorption capacity, calculated by applying the Langmuir model (12.08 mg·g^−1^), was consistent with the uptake capacity estimated using isotherm experiments (9.9 mg·g^−1^).

### 3.4. Kinetics of Chromium (VI) Retention

The main steps of SPE and/or d–*μ* SPME are the transfer of the analyte from the aqueous solution to the sorbent/adsorbent surface, and then the passage of the analyte species into the interior of the solid pores and membranes [82, 83]. Hence, assigning the kinetics of chlorochromate anion (CrO3Cl^−^) sorption by the developed sol-gel/PQ^+^.Cl^−^/PUFs microextractor is of great significance for the minimization and/or the complete removal of chromium (VI). The primary study of chromium (VI) sorption from the test aqueous HCl solution by the developed sol-gel/PQ^+^.Cl^−^/PUFs microextractor at the optimized parameters was found to be fast and dependent on shaking time as it reached equilibrium within a ∼50–60 min shaking period. The value of the half-life time (*t*_1/2_) of sorbed chromium (VI) was low (2.8 ± 0.028 min). Therefore, the data were further subjected to many kinetic models including nonlinear [50, 83] and linear models such as Weber–Morris [85, 86], pseudofirst order (Lagergren) [86, 87], pseudosecond order [88], and Elovich [89].

**Figure 3:**
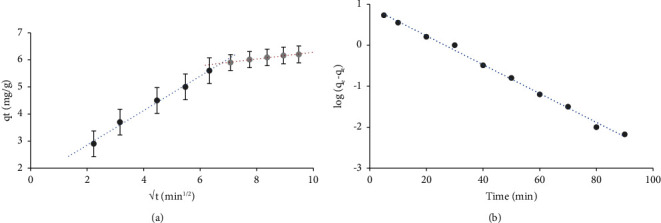
The kinetic model plots of chromium (VI) retention at 25 ± 1°C: Weber–Morris (a) and Lagergren model (b).

The impact of time was further studied by the Weber–Morris model [85] to assign whether intraparticle diffusion or film diffusion is the rate governing step in the chromium (VI) uptake by sol-gel/PQ^+^.Cl^−^/PUFs. The most common Weber–Morris model can be stated by the following equation:

(13) $$\begin{array}{c} q_{t}=R_{d}{\left(t\right)}^{\left(1/2\right)}+C, \end{array}$$

where *R*_*d*_ is the rate constant of intraparticle transport (mg·g^−1^·min^−1/2^), *q*_*t*_ is the equivalent quantity of retained analyte (mg·g^−1^) at a specific time *t,* and *C* (mg·g^−1^) is the thickness of the boundary layer, which is the intercept from the initial linear portion of the linear plot, where a higher value of C has a greater effect on the boundary layer [85]. The plot of *q*_*t*_*versus* the square root of time was found to be linear (*R*^2^ = 0.9929) up to 45 ± 1 min and deviates on intensifying shaking time (Figure 3(a)). The linear plot at the early stage does not pass through the origin, which confirms that the rate-controlling step for sol-gel/PQ^+^.Cl^−^/PUFs is not only intraparticle diffusion [87]. The fact that at the initial stage of shaking time, the diffusion rate of the chromium (VI) species on the established solid-phase extractor was high and diminished linearly on increasing shaking time with a correlation coefficient (*R*^2^) of 0.9842. Thus, it can be concluded that the rate-controlling step of chromium (VI) sorption is film diffusion at the initial stage of extraction [86, 87]. In the second stage of chromium (VI) extraction, the intraparticle diffusion decreased, indicating that the pore volumes of the sorbent are most likely exhausted [86, 87].

The presence of different pore sizes could be a reason behind the change in *R*_*d*_ values of the first and subsequent stages from 0.6378 to 0.1251 mg·g^−1^·min^−1/2^, as calculated from the slopes (Figure 3(a)) [85, 86]. Depending on the high value of the constant C (1.5755 mg·g^−1^), it was considered that film diffusion plays a major role in the rate-controlling step in the overall chromium (VI) uptake and the intraparticle diffusion step cannot be the rate-controlling step [86, 87]. Moreover, the first stage occurred rapidly after transferring the chromium (VI) species from the extraction solution, i.e., the uptake stages include transport of the chromium (VI) species from the bulk liquid phase to the external surface of the d–*µ* SPME through a hydrodynamic boundary layer or film solution (film diffusion), and then the diffusion of the chromium (VI) species from the exterior of the SPE (external diffusion) as reported for anionic dyes onto hollow polymer microcapsules [87, 89].

The data were further analysed by the pseudofirst-order (Lagergren) model [86] as

(14) $$\begin{array}{c} \log\left(q_{e}-q_{t}\right)=\log\;q_{e}-\frac{k_{1}}{2.303}t, \end{array}$$

where *k*_1_ (min^−1^) is the first-order rate constant for chromium (VI) sorption and *q*_*e*_ and *q*_*t*_ (mg·g^−1^) are the quantities of sorbed chromium (VI) per unit mass of the established solid extractor at equilibrium (*q*_*e*_) and at time *t* (*q*_*t*_), respectively. The plot of log (*q*_*e*_ − *q*_*t*_) *versus* time was linear (Figure 3(b)) with a correlation coefficient of (*R*^2^ = 0.9961). The calculated value of the first-order rate constant *k*_1_ was 0.081 min^−1^. The equilibrium capacity (*q*_*e*_) for chromium (VI) sorption from the intercept of the linear plot of log (*q*_*e*_ − *q*_*t*_) *versus* time was found to be equal to 8.6 mg·g^−1^, which is in good agreement with the experimental value (9.9 mg·g^−1^).

The results were additionally analysed by the pseudosecond-order model [87] as

(15) $$\begin{array}{c} \frac{t}{q_{t}}=\frac{1}{h}+\left(\frac{1}{q_{e}}\right)t, \end{array}$$

where *h* *=* *k*_2_*q*_*e*_^2^ can be assumed as the initial rate constant of the sorption step and *k*_2_ is the pseudosecond-order rate constant g(mg^−1^·min^−1^). The plot of *t*/*q*_*t*_*versus* time for chromium (VI) uptake by the established solid extractor was found to be linear, as shown in ESI.10, with an acceptable correlation coefficient (*R*^2^ = 0.9984). The equilibrium capacity (*q*_*e*_) of chromium (VI) retention, as calculated from the slope of the linear plot, was found to be 6.75 mg·g^−1^; whereas the pseudosecond-order rate constant (*k*_2_) was found to be equal to 0.017 g (mg^−1^·min^−1^). Furthermore, the disagreement between the calculated *q*_*e*_ value from the pseudosecond-order model (6.75 mg·g^−1^) and the experimental *q*_*e*_ value (9.9 mg·g^−1^) indicates that the chromium (VI) sorption by the established solid microextractor does not follow the pseudosecond-order model. The data evidently display that the pseudofirst-order model is properly fitted for chromium (VI) sorption kinetics [87].

**Figure 4:**
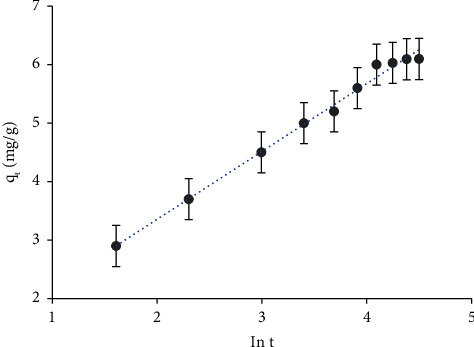
Plot of the Elovich kinetic model for chromium (VI) retention onto sol-gel/PQ^+^.Cl^−^/PUFs at pH < 1 at 25 ± 1°C.

The data were further subjected to McLintock–Elovich kinetic mode [88]. This model is frequently used for systems in which the surface of the solid sorbent is heterogeneous and applicable mainly for chemisorption kinetics. Elovich kinetic mode can be stated by the following linear equation:

(16) $$\begin{array}{c} q_{t}=\frac{1}{\beta }\ln\left(\alpha \beta \right)+\frac{1}{\beta }\ln\;t, \end{array}$$

where *α* (mg·g^−1^·min^−1^) is the initial sorption rate and *β* (mg·g^−1^) is the desorption constant linked to the extent of the surface coverage and activation energy for chemisorption.  Figure 4 shows the plot of *q*_*t*_*versus* ln *t*. The plot was linear at the early stage of shaking time (ln *t* < 4.1 min) with a correlation coefficient (*R*^2^) of 0.9918. The *α* and *β* parameters of Elovich were safely calculated from the intercept (1/*β* ln (*α β*)) and slope (1/*β*) of Figure 4. The computed values of the Elovich parameters *β* and *α* were found to be equal to 0.8619 mg·g^−1^ and 2.83 mg·g^−1^·min^−1^, respectively.

### 3.5. Thermodynamic Parameters of Chromium (VI) Sorption

**Figure 5:**
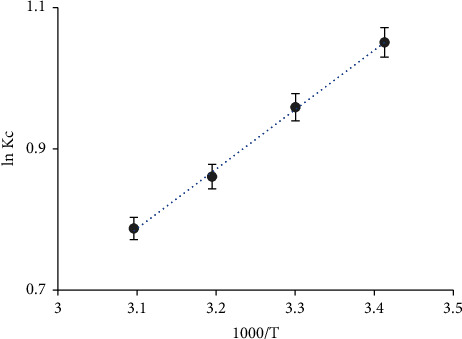
Plot of ln *K*_*c*_*versus* 1000/*T* for chromium (VI) sorption from an aqueous HCl solution (pH < 1) onto sol-gel/PQ^+^.Cl^−^/PUFs after 60 min shaking time.

In SPE or d–*µ* SPME, the impact of the changes of the thermodynamic parameters enthalpy (Δ*H*), entropy (Δ*S*), and Gibbs free energy (Δ*G*) is of great importance. Thus, the sorption of the chromium (VI) species from the aqueous solution by the established sol-gel/PQ^+^.Cl^−^/PUFs microextractor at a pH < 1 was studied over a wide range of temperatures (293–333 K). Considering no complex formation and/or precipitation of CrO_3_Cl^−^ species took place, and the tested species is present as a neutral species at the optimized pH < 1, the thermodynamic parameters Δ*H*, Δ*S*, and Δ*G* of chromium (VI) sorption onto the used sorbent can be computed from the linear plot of ln *K*_*C*_*versus* 1000/*T* (Figure 5), where *K*_*C*_ is the value for analyte retention depending on the fractional attainment (*F*_*e*_) of the sorption process (*K*_*C*_ = *F*_*e*_/1 − *F*_*e*_). The plot was linear over the entire temperature range with an acceptable correlation coefficient (*R*^2^ = 0.9985). The equilibrium constant increased on growing temperature, revealing that the chromium (VI) retention onto the established solid extractor is an endothermic process [90]. The numerical values of Δ*H*, Δ*S*, and Δ*G* as evaluated from the slope and intercept of the linear plot were found to be equal to 6.99 kJ·mol^−1^, 15.13 kJ·mol^−1^·K^−1^, and −8.14 kJ·mol^−1^ (at 293 K), respectively.

Considering Van't Hoff equation, the distribution coefficient (*D*) of chromium (VI) retention is linked to absolute temperature (*T*) according to the following equation:

(17) $$\begin{array}{c} \log\;D=\frac{-∆H}{2.30\text{ RT}}+C. \end{array}$$

The plot of log *D versus* 1000/*T* for chromium (VI) sorption onto the solid sorbent was linear (ESI.11). The *D* values of chromium (VI) sorption from the aqueous media with pH < 1 onto the established solid platform increased on growing temperature, revealing the endothermic nature of the retention step [91]. The value of Δ*H* of chromium (VI) uptake onto the solid sorbent as calculated from ESI.11 was found to be equal to 6.97 ± 0.3 kJ·mol^−1^, which agrees with the data calculated from Figure 5 (6.99 kJ·mol^−1^), confirming the endothermic nature of chromium (VI) uptake.

The reaction follows a physical sorption mechanism since the Δ*H* value (6.97 ± 0.3 kJ·mol^−1^) is lower than the expected value of chemical sorption 10 kJ·mol^−1^. The sorption capacity of the established sorbent increased on growing temperature. This trend is most likely attributed to the bonds' strength between chromium (VI) and the sorbent's active sites with increasing temperature. The Growing temperature may control the physical characteristics of the sol-gel/PQ^+^.Cl^−^/PUFs sorbent and enhances the intermolecular interactions between the sorbent and analyte. At the studied temperatures, the Δ*G* value indicated the feasibility and spontaneous nature of chromium (VI) uptake on increasing temperature. Furthermore, the decrease in Gibbs free energy (Δ*G*) with increasing temperature implies that the chromium (VI) sorption process is spontaneous, endothermic and is more favourable at a high temperature. The energy of the active sites of the sorbent provided by raising the temperature most likely enhances the possible interaction between the active sites of the established sol-gel/PQ^+^.Cl^−^/PUFs and the oxychromium (VI) ions resulting in a higher sorption percentage of the analyte.

### 3.6. Mechanism of Chromium (VI) Sorption

Based on the sorption characteristics, kinetics, and thermodynamic parameters of chromium (VI) uptake by the established sol-gel/PQ^+^.Cl^−^/PUFs sorbent from an aqueous solution of pH < 1, it can be recognized that the CrO_3_Cl^−^ uptake is most likely attributed to a multimode sorption mechanism involving (i) absorption related to “weak anion exchange” and/or “solvent extraction” of the binary complex ion associates {[–CH_2_–OH^+^–CH_2_–].[CrO_3_Cl]^−^}_sol-gel/PUFs_, {[–NH_2_ + –COO–].[CrO_3_Cl]^−^}_sol-gel/PUFs,_ and{[PQ]^+^.[CrO_3_Cl]^−^}_sol-gel/PUFs_; (ii) H-bonding between the silanol group of the sol-gel/PUFs and the bulky anion [CrO_3_Cl]^−^; and (iii) the electrostatic *π*–*π* interaction, which resulted between CrO_3_Cl^−^ and both the silanol group (Si/ZrO_2_ and Si–O–Zr) and siloxane (Si–O–Si) groups of the sol-gel/PUFs [92]. All these processes most likely participated in the CrO_3_Cl^−^ absorption. On the other hand, “surface adsorption” on/in the sol-gel/PUFs membrane [56] may also participate in the CrO_3_Cl^−^ sorption. Thus, the overall sorption mechanism of chromium (VI) by the established sol-gel/PQ^+^.Cl^−^/PUFs is most likely expressed by the following equation [62, 78]:

(18) $$\begin{array}{c} C_{r}=C_{\text{abs}}+C_{\text{ads}}=DC_{\text{aq}}+\frac{SK_{L}C_{\text{aq}}}{1+K_{L}C_{\text{aq}}}, \end{array}$$

where *C*_*r*_ and *C*_aq_ are the equilibrium concentration of chromium (VI) onto the solid sorbent and the remaining concentration in the aqueous solution, respectively. The parameters *C*_abs_ and *C*_ads_ are the equilibrium concentration of chromium (VI) onto the sorbent as an absorbed and adsorbed species, respectively. *S* and *K*_*L*_ are the saturation values for the Langmuir adsorption.

These result in addition to the resilience characteristics of the PUFs, sol-gel coating technology may also enrich the porous structure of the PUFs, ensuing an improvement in the extraction efficiency of the CrO_3_Cl^−^ anion via polycondensation of the residual hydroxyl Si–OH and Zr–OH into hydrophobic Si–O–Si and Si–O–Zr groups of the sol-gel as reported [92]. These results suggest the use of the established sol-gel-treated PUFs microextractor in column mode for complete removal and recovery of oxychromium (VI) species in environmental water samples.

### 3.7. Interference Study

The developed extractor towards CrO_3_Cl^−^ (10.0 *µ*g·mL^−1^) sorption from the water was studied via the batch mode of separation in the presence of a series of coexciting inorganic ions, which are commonly present in environmental water samples. The tolerance limit (w/v) of less than ±5% change in the extraction percentage of CrO_3_Cl^−^ was considered free of interfering species. The impact of the cations NH_4_^+^, Na^+^, K^+^, Cr^3+^, Cu^2+^, Zn^2+^, Al^3+^, and Ni^2+^, as well as the anions NO_3_^−^, CH_3_COO^−^, F^−^, Cl^−^, and Br^−^ at a high mass excess (50-fold) was tested. The data revealed a nonsignificant interference of these ions on chromium (VI) uptake. These results added further support to the utility of the developed extractor towards the removal of chromium (VI) from environmental water samples.

## 4. Analytical Applications

### 4.1. Chromium (VI) Retention and Recovery (*R*) by the Sorbent-Packed Minicolumn Mode

The proposed sol-gel/PQ^+^.Cl^−^/PUFs were successfully implemented for complete enrichment and recovery of standard concentrations (0.01–1.0 *µ*g·mL^−1^) of chromium (VI) spiked into deionized water and tap water samples as described above. The results revealed complete sorption of chromium (VI), as indicated from the absorbance of the total effluent solution. The sorbed chromium (VI) species on the sol-gel/PQ^+^.Cl^−^/PUFs were then recovered quantitatively from the sorbent-packed column. The analytical results for chromium (VI) spiked into deionized water and tap water are summarized in Table 2. These results confirm the analytical utility of the established solid sorbent for the complete removal and recovery of chromium (VI) species from water samples at a reasonable flow rate.

### 4.2. Chromium (VI) Retention and Recovery (*R*) by the Established Sorbent Packed Pulse Column

In this experiment, a series of prefiltered tap water samples spiked with known concentrations (0.01–1.0 *µ*g/mL) of chromium (VI) at the optimized parameters of extraction were extracted by the sol-gel/PQ^+^.Cl^−^/PUFs packed medical syringe (0.2 ± 0.01 g) as a pulse column, as described above. By 25–30 pulses, complete retention (98 ± 2.6%) of the chromium (VI) species was achieved, as indicated by the absorbance of the test acidic aqueous solution after extraction at *λ*_max_ against the reagent blank (Table 3).

The extraction efficiency (*E*_*n*,op_) in the open arrangement is related to the distribution ratio (*K*_*D*_), the number of pulses (*n*), and the maximum volume concentration (*p*) (*p* = *W*_0_/*V*_*P*_), where W_0_ is the total sample volume and *V*_*P*_ is the compressed foam volume as expressed by the following equation:

(19) $$\begin{array}{c} E_{n,\text{op}}=\frac{K_{D}}{P}\left[1-{\left(\frac{{nK}_{D}}{P+nK_{D}}\right)}^{n}\right]. \end{array}$$

The retained chromium (VI) species onto the sorbent-packed pulse was then completely recovered from the sorbent packed pulse column using NaOH (20 mL, 0.5 M) for 25–30 pulses, as indicated from the absorbance of the acidic test solution at 355 nm. The results are summarized in Table 3 and confirm the good performance of the established d–*µ* SPME towards complete extraction and recovery of chromium (VI) from environmental water samples. The data added further support the utility of the solid sorbent for the complete removal and recovery of chromium (VI) species from water samples by a pulse-packed column.

## 5. Conclusion and Future Perspective

In summary, the current study reports a facile preparation of sol-gel-functionalized polyurethane foams as an effective dispersive solid-phase microextractor for complete removal and subsequent determination of chromium (VI). The treated foams can serve as an advanced dispersive solid-phase microextractor due to their attractive resilience, flexibility, adjustable surface characteristics, and great capability to extract a series of inorganic and organic species from an aqueous solution. Nanosized sol-gel provides high surface area, H-bonding, and specific affinity for analyte retention on a solid-phase extractor such as PUFs. The membrane-like structure of PUFs solid sorbent facilitates its analytical utility as an effective and resilient solid platform sorbent material for the complete sorption of chromium (VI) species from an aqueous media. In future studies, the established extractor can be used for the complete extraction of chromium (III) and chromium (VI) after the complete oxidation of the former species with H_2_O_2_ in an alcoholic KOH solution.

## Figures and Tables

**Table 1:** Adsorption capacities of different adsorbents for removal of Cr (VI) ions from water samples.

Adsorbent/sorbent	Shaking time (min)	*q*_*e*_ (mg·g^–1^)	Ref
Red mud	1440	75	Gupta et al. [65]
MWCNT	—	9.5	Tuzen and Soylak [66]
Pomegranate peel	—	9.45	Mohammed et al. [67]
Chitosan	—	7.94	Aydın and Aksoy [68]
Rich husk ash	180	25.64	Bhattacharya et al. [69]
Grafted macadamia nutshell powder	180	39.21	Ntuli and Pakade [70]
Banana peel	60	10.42	Parlayici and Pehlivan [71]
Pleurotus mutilus biomass	180	15.50	Alouache et al. [72]
Sol-gel/PQ^+^.Cl^−^/PUFs	50	9.9	*Present study*

**Table 2:** Recovery percentage (%) of chromium (VI) spiked into deionized and tap water samples by the established sorbent-packed minicolumn at a 10.0 mL/min flow rate.

Matrix	Chromium (VI) added (*µ*g/mL)	Chromium (VI) found (*µ*g/mL)	Recovery (%)
Deionized water	0.01	0.01 ± 0.00004	100 ± 0.4
	0.10	0.1 ± 0.0005	100 ± 0.5
	1.0	0.95 ± 0.004	95 ± 0.4

Tap water	0.01	0.01 ± 0.00008	100 ± 0.8
	0.10	0.1 ± 0.0006	100 ± 0.6
	1.0	0.96 ± 0.004	96 ± 0.4

**Table 3:** Recovery percentage of chromium (VI) spiked into tap water samples by the established sorbent-packed pulse column (0.2 ± 0.001 g) after 25–30 pulses.

Chromium (VI) added (*µ*g/mL)	Chromium (VI) found (*µ*g/mL)	Recovery (%)
0.01	0.01 ± 0.00008	100 ± 0.8
0.05	0.05 ± 0.00035	100 ± 0.7
0.50	0.5 ± 0.003	100 ± 0.6
1.0	0.95 ± 0.006	95 ± 0.6

## Data Availability

Electronic supporting information (ESI) will be available online.

## Supplementary Materials

Supplementary Materials Table 1 shows the operational conditions of ICP-OES of chromium determination, while scheme 1 and the next four figures show the preparation and characterization of the sol-gel. Figures 7 to 11 show the relation between chromium retention and different parameters such as time, temperature, and concentration.
