# Supplementary material for: An Effective Sol-Gel-Functionalized Polyurethane Foams Solid Platform Packed Minicolumns for Complete Extraction of Chromium (VI) from Water: Kinetic, Sorption Isotherms, Thermodynamic Study, and Analytical Utility
Source: Int J Anal Chem. 2024 Aug 6;2024:3152894. doi: 10.1155/2024/3152894 (PMC11458274; doi:10.1155/2024/3152894)
Supplement: Supplementary Materials — Table 1 shows the operational conditions of ICP-OES of chromium determination, while scheme 1 and the next four figures show the preparation and characterization of the sol-gel. Figures 7 to 11 show the relation between chromium retention and different parameters such as time, temperature, and concentration. [file 3152894.f1.docx]

An Effective Sol-gel functionalized polyurethane foams solid platform packed mini-columns for complete extraction of chromium (VI) from water: kinetic, sorption isotherms, thermodynamic study and analytical utility

**Effat A. Bahaidarah**

Department of Chemistry, Faculty of Science, King Abdulaziz University, P. O. Box 80203, Jeddah 21589, Saudi Arabia

**^*^**Corresponding author, e-mail: [ebahaidarah@kau.edu.sa](mailto:ebahaidarah@kau.edu.sa)
ORCID ID :0000-0003-2502-0916

**Electronic Supplementary Information (ESI)**

ESI.1 Table 1 ICP- OES operational conditions for chromium determination

| **Parameter** | **Unit** |
| --- | --- |
| Rf Power | 1400W |
| Nebulizer Flow | 0.7 L/min |
| Auxiliary Flow | 0.3 L/min |
| Plasma Flow | 10.0 L/min |
| Sample Pump Flow | 1 mL/min |
| Plasma Viewing | Axial |
| Processing Mode | Area |
| Replicates | 3 |
| Nebulizer Type | Cross-flow (Gim Tip) |
| Spray Chamber | Scott (Ryton) |
| Injector | Scott (Ryton) |
| Wavelength | Cr 267.7, nm |


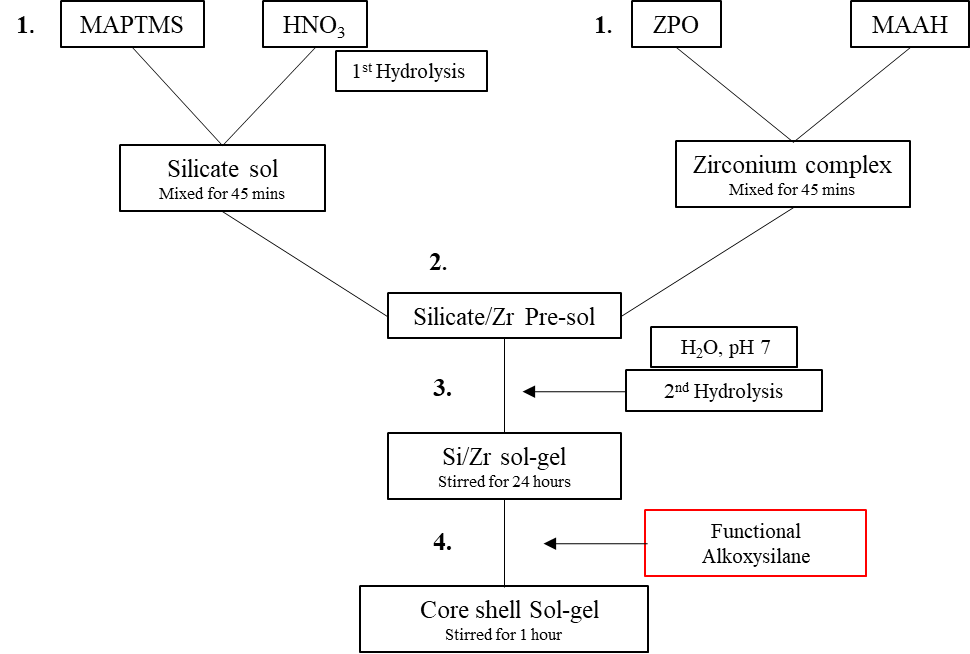


**ESI. 2** **Scheme 1.** A flow chart describing the four steps of the preparation of the reference and functional Sol-gel materials (MacHugh et al. 2019).

**
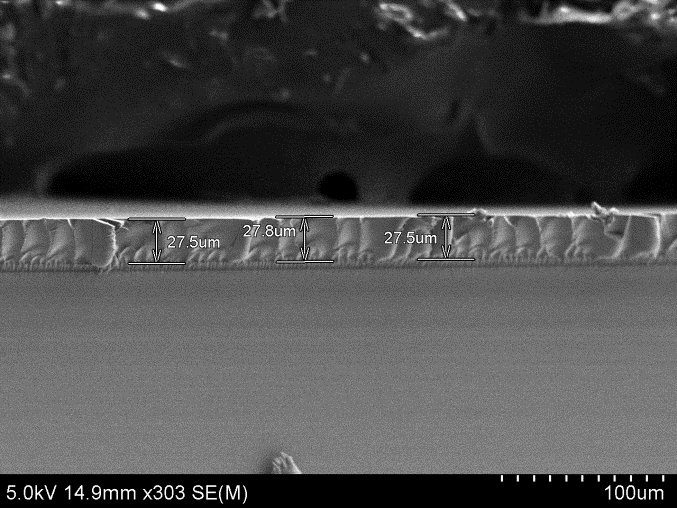
**

**
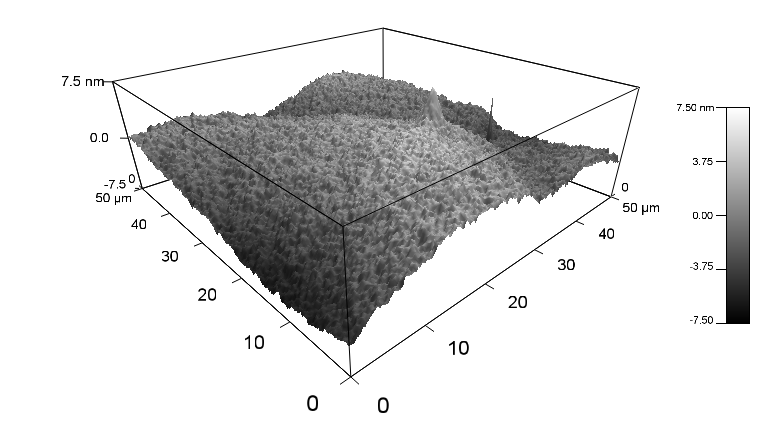
ESI.3** SEM image of the hybrid Sol-gel solution in isopropanol.

ESI.4 AFM image of the Sol-gel solution.

ESI.5 EDX analysis of the hybrid Sol-gel solution.

A

B

ESI.6 FT-IR spectra of the Sol-gel in the range (A) 500-4000 and (B) 800-1250 cm^-1^.

ESI.7 Plot of distribution ratio (D) *versus* shaking time of chromium (VI) retention in HCl media (1.0 M) onto Sol-gel/ PQ^+^.Cl^-^ / PUFs at 25±1 ^o^C.

ESI.8 Plot of the distribution ratio (D) *versus* solution temperature (20-50 ^0^C) of chromium (VI) retention onto Sol-gel/ PQ^+^.Cl^-^ / PUFs at pH<1 after 50 min shaking time.

**ESI.9** Plot of distribution ratio (D) of chromium (VI) sorbed onto Sol-gel**/** PQ^+^.Cl^-^ **/** PUFs at pH<1 after 60 min shaking time.

ESI.10 Pseudo-second-order kinetic plot for chromium (VI) retention onto Sol-gel/ PQ^+^.Cl^-^ / PUFs at pH <1 and 25±1 ^o^C

ESI.11 Van’t Hoff plot for chromium (VI) sorption from the aqueous solution of pH <1 after 60 min shaking time
